# Lipoprotein(a) levels in Irish subjects from a specialised lipid centre

**DOI:** 10.1007/s11845-025-04003-5

**Published:** 2025-08-09

**Authors:** Iulia Tustiu, Dilara Ensar, Ailish O’Keeffe, Eoin Begley, Gerard Boran, Richard Armstrong, Vincent Maher

**Affiliations:** 1 Cardiology Department, Tallaght University Hospital, Dublin, Ireland; 2 Mater Misericordiae University Hospital, Dublin, Ireland; 3 General Medicine Department, Tallaght University Hospital, Dublin, Ireland; 4 Department of Laboratory Medicine, Tallaght University Hospital, Dublin, Ireland; 5 Department of Clinical Chemistry, Tallaght University Hospital, Dublin, Ireland; 6 School of Medicine, Trinity College Dublin, Dublin, Ireland

**Keywords:** Aortic stenosis, Atherosclerosis, Cardiovascular risk, Irish cohort, Lipidaemic subgroups, Lipoprotein (a)

## Abstract

**Background:**

Lipoprotein(a) is a low-density lipoprotein-like particle covalently bound to apolipoprotein(a). It exhibits pro-atherogenic and pro-inflammatory effects and is an established independent monogenic determinant of atherosclerotic cardiovascular disease and aortic valve stenosis [1–4].

**Aims:**

To establish the Lp(a) distribution in a native Irish population and to explore if a certain lipid profile was associated with high Lp(a) level.

**Methods:**

We retrospectively included all subjects with Lp(a) results tested in our laboratory between January 2021 and December 2022. Patients were divided into Irish and non-Irish name subgroups [16]. We analysed the Lp(a) distribution across lipidaemic subgroups. Statistical analyses were completed in Jamovi programme V2.3.26.

**Results:**

In total 2762 patients of which 1899 had also a lipid profile. Eighty-five percent (*n* = 2359) of individuals had Irish surnames and 60% (*n* = 1419) were males. Mean age of all patients was 56 ± 17 years. The median lipoprotein(a) level was 34.5 nmol/L (interquartile interval < 20 to 153). The Lp(a) median in females was 37.3 (interquartile interval < 20 to 169) versus males 32.9 (interquartile interval < 20 to 147) (*p* = 0.029). A total of 62.9% (*n* = 1738) of Irish subjects had Lp(a) levels < 75 nmol/L, 7.56% of them (*n* = 209) between 75 and 125 nmol/L and 29.5% (*n* = 815) of subjects had Lp(a) > 125 nmol/L.

**Conclusions:**

This is the largest study of Lp(a) distribution in an Irish population revealing positively skewed Lp(a) serum levels. This is not entirely reflective of the general population but brings to the fore the additional hidden high risks in those patients attending cardiovascular services. More education is needed to increase the use of Lp(a) measurements and guide further therapy.

## Introduction

Lipoprotein(a) (Lp(a)) is a low-density lipoprotein (LDL)-like particle covalently bound to apolipoprotein(a). It exhibits pro-atherogenic and pro-inflammatory effects and is an established independent monogenic determinant of atherosclerotic cardiovascular disease (ASCVD) and aortic valve stenosis [1–4]. Lp(a) levels vary widely across ethnic groups [5, 6]. Higher levels are associated with an increased ASCVD risk, and this risk is amplified by the presence of concomitant traditional risk factors and diminished by LDL cholesterol reductions [7–14].

The European Atherosclerotic Society panel’s consensus is to recommend Lp(a) screening at least once in an adult’s lifetime, especially in populations with a predisposition for premature ASCVD [11]. This would enhance early identification of high-risk individuals, risk re-stratification, and commencement of tailored primary prevention [15].

Screening for high Lp(a) plasma levels has been an integral aspect of lipid management in the Advanced Lipid Management and Research (ALMAR) Centre. In an effort to assess Lp(a) levels in a typical population referred for cardiovascular disease assessment, we analysed all patients who had an Lp(a) level measured over the course of 2 years at the ALMAR centre. Our primary goal was to establish the Lp(a) distribution in a native Irish population referred for cardiovascular disease assessment. Secondly, we explored whether or not a certain lipid profile was associated with the presence of high Lp(a) to help guide appropriate screening for this risk factor.

## Methods

We retrospectively included all subjects (*n* = 2762) with Lp(a) results from our hospital laboratory between 1 st of January 2021 and 31 st of December 2022. We included the first recorded Lp(a) result and excluded duplicate values.

Patient ethnicity was established based on surnames [16] and were divided into Irish and non-Irish name subgroups accordingly.

We also analysed the Lp(a) distribution across lipidaemic subgroups, with normal lipidaemic profiles (group 1) being defined by triglyceride < 1.7 mmol/L, HDL (females) > 1.2 mmol/L, HDL (males) > 1 mmol/L, LDL ≤ 3 mmol/L; hyperlipidaemia (group 2) with LDL > 3 mmol/L alone (i.e. triglyceride < 1.7 and HDLc in normal range); and atherogenic dyslipidaemia (group 3) with triglyceride > 1.7 mmol/L, HDL (females) < 1.2 mmol/L, and HDL (males) < 1 mmol/L [15].

Additional data collected included age, gender, ethnicity, and full lipid profile (total cholesterol, HDL, triglyceride, and LDL) when available at time of Lp(a) sampling.

Tests for the full lipid profile (total cholesterol, HDL-cholesterol, triglyceride) were analysed in the Clinical Chemistry Department at Tallaght University Hospital on the Roche COBAS® Modular system using (fasting) lithium heparin plasma samples. The laboratory holds ISO-15189 accreditation and maintained satisfactory performance in the RIQAS external quality assurance scheme for Lp(a) and lipid profile assays during the study period. The COBAS® Tina-quant Gen.2 lipoprotein (a) assay is calibrated using the SRM2B International Reference Reagent recommended by the WHO/IFCC. This allows apo(a) size-independent determination of Lp(a) molar concentrations which can be reported in nmol/L (nanomoles/litre) as recommended by the EAS Consensus Panel [11]. Moreover, the assay has excellent total and within-run precision around the clinical decision points of 75 and 125 nmol/L. LDL-cholesterol was calculated in suitable samples using the Friedewald formula.

Statistical analyses were performed using the Jamovi programme Version 2.3.26. We employed descriptive statistics to present patient demographics. Chi square and Fisher’s ANOVA were used to compare the Lp(a) medians among lipid subgroups. We used a Spearman test to examine the correlation between Lp(a) (nonparametric) and other lipid measurements.

## Results

A total of 2762 eligible patients were included of which 1899 had also a full lipid profile performed within our laboratory over the same period of time (Table 1). Of the total cohort analysed, we identified 85% (*n* = 2359) of individuals with Irish surnames and 60% (*n* = 1419) of whom were males. The mean age of all patients was 56 ± 17 years.

**Table 1:** Demographics and lipids serum levels

	***Total population***	***Irish names***	***Non-Irish names***
***Lp(a) individuals***	**n=2762**	**n=2359 (85%)**	**n=403 (15%)**
*Age years (mean±SD)*	56±16	57.2±16	48.5±14
*Gender (males, %)*	n=1663, 60%	n=1419, 60%	n=244, 60%
*Lp(a) (median, IQR) (nmol/L)* *Genders (median)*	34.5 (<20-153) M (32.9) F (37.2)	34.6 (<20-156) M (33.1) F (36.5)	34 (<20-139) M (30.6) F (42.4)
***Full lipid profile individuals (n,%)****(mmol/L)*	**n=1899**	**n=1630 (86%)**	**n=269 (14%)**
*Total Cholesterol (mean±SD) *	4.50 ±1.43	4.43 ±1.41	4.92±1.48
*LDL (mean±SD) *	2.59 ±1.24	2.52±1.22	3.02 ± 1.27
*HDL (mean± SD) *	1.24± 0.41	1.24±0.42	1.21±0.38
*Triglycerides (median, IQR)*	1.32, (0.94-1.87)	1.31, (0.93- 1.84)	1.38 (0.94-2.18)

The median lipoprotein(a) serum level was 34.5 nmol/L (interquartile interval < 20 to 153), with no significant differences noted between Irish (median, range) and non-Irish names cohorts (median, range). The Lp(a) median in females was higher, 37.3 (Interquartile interval < 20 to 169) versus males 32.9 (interquartile interval < 20 to 147) (*p* = 0.029). Lp(a) levels were positively skewed across all subgroups—names, origin, and genders.

**Fig. 1:**
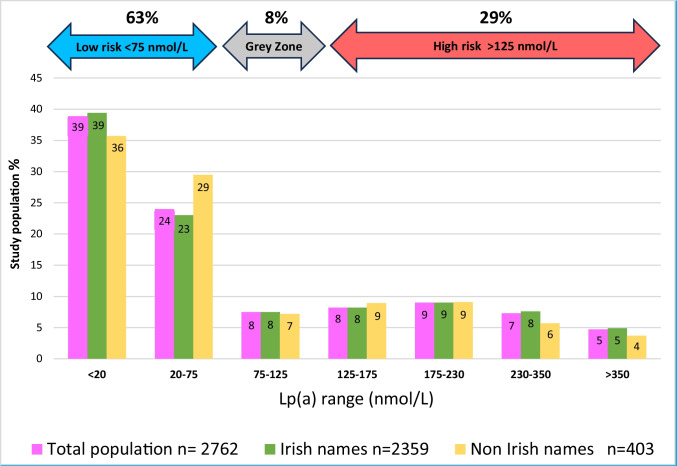
Lp(a) serum level distribution in Ireland’s population

By extrapolating our population Lp(a) level results to the cut offs of Lp(a) serum ranges proposed by Kronenberg et al. [8], we found that 62.9% (*n* = 1738) of the Irish subjects had Lp(a) levels < 75 nmol/L with a low ASCVD risk, 7.56% (*n* = 209) of subjects had Lp(a) levels between 75 and125 nmol/L in the grey zone of ASCVD risk, and 29.5% (*n* = 815) had Lp(a) plasma levels above 125 nmol/L with high ASCVD risk (Fig. 1).

We also compared our Irish subjects results with Lp(a) quartile cut-offs of a larger Caucasian cohort [17]. This revealed that a greater proportion of Irish subjects (41%) had Lp(a) levels in the highest quartile of the Danish cohort quartile cut-offs (Table 2). However, the majority (93%) of the Irish subjects were tested within a hospital context and the results are therefore not representative of the general population of Ireland.

**Table 2:** Proportion of Irish population in ranges derived from Danish population

*Quartiles*	*Danish population* *Lp(a) ranges* *(nmol/L)*	*Danish population percentages*	*Irish population* *percentage*
* 1 st Q*	<6.4	25%	39%
*2nd Q*	6.4-17	25%	
*3rd Q*	18-59	25%	20%
*4th Q*	>59	25%	41%

Of the total number of Lp(a) tests performed at the specialist lipid (ALMAR) centre over the 2-year period, only 5% (*n* = 141) were requested by primary care service (i.e. reflective of a general population), with 26% (*n* = 37) of these individuals being found to have high-risk Lp(a) levels (> 125 nmol/L).

On further data analysis, we identified a total of 1899 individuals of the total cohort who also had a full lipid profile completed in our laboratory over the same period of time (Table 1).

There was a weak but significant association between lipoprotein(a) levels and total cholesterol (*r* = 0.077), LDLc (*r* = 0.065), HDLc (*r* = 0.079), and triglycerides (r =  − 0.063) (all *p* ≤ 0.006), respectively.

**Fig. 2:**
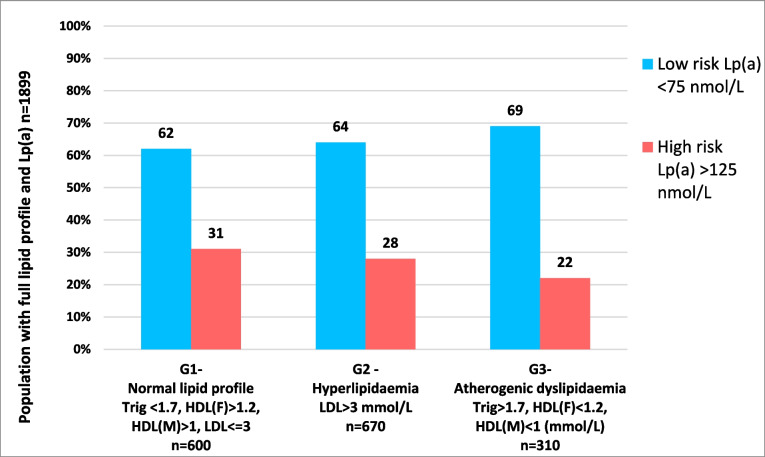
Irish population distribution with low and high-risk Lp(a) serum levels across lipidaemic subgroups

We also examined the Lp(a) distribution for these Irish subjects across the defined lipidaemic subgroups. Figure 2 displays the proportion of individuals with high-risk Lp(a) (> 125 nmol/L) and low-risk Lp(a) (< 75 nmol/L), across the three lipidaemic subgroups. Although there was no statistical significant difference in the Lp(a) distribution across lipidaemic subgroups, there was a trend for a greater proportion of low Lp(a) levels and a smaller proportion of high-risk Lp(a) levels in the atherogenic dyslipidaemia subgroup.

## Discussions

The Lp(a) distribution in this population of mainly Irish subjects attending a specialised cardiovascular clinic is positively skewed and displays a significant gender difference which follows a similar pattern to other larger cohorts [17, 18].

In this study, 63% of individuals had low risk levels of Lp(a) (< 75 nmol/L) and 29% are classified as high-risk for ASCVD based on Lp(a) serum levels > 125 nmol/L. When this Irish cohort was compared with a larger north European population, the UK biobank [19], it revealed that a higher proportion of Irish people (21% versus 9.2%) had very high-risk Lp(a) levels > 175 nmol/L. Moreover, when compared to a Danish general population [17], our Irish subjects displayed a higher proportion with high levels Lp(a) (41% versus 25% of the 4th Danish quartile). However, the UK and Danish studies involved a general population [17, 19], whereas our study included mainly (approximately 93%) hospital-attending individuals. Although, our results are not directly representative of the Irish general population, they do represent the Lp(a) values of those referred to a general cardiology service.

In an effort to determine if there was a lipidaemic subgroup of patients more likely to have a high Lp(a) level and hence guide a preferential Lp(a) sampling subgroup, we assessed for this relationship. However, while Lp(a) levels did vary with lipidaemic subgroups to a small extent, there was no significant difference to guide a clinical practice change to select those likely to have high Lp(a) levels. As previously observed, we also found that those who had an atherogenic dyslipidaemic pattern typical of diabetes and insulin resistance [20] had a greater proportion of patients with low Lp(a) levels.

Lp(a) is an established strong, independent, and monogenic risk factor for ASCVD, and its cardiovascular disease impact is amplified in the presence of other traditional risk factors, especially lipid abnormalities [11]. Given that there have been a number of studies highlighting that recommended lipid targets [21] are not being achieved in Irish populations with or without proven ASCVD [22, 23] and the fact that our study revealed high Lp(a) levels in Irish subjects adds further concern regarding the lack of lipid target achievement in this country.

If as recommended that Lp(a) measurements are undertaken at least once in everybody’s lifetime, we recommend that this is done in early life so that the added weight of a high Lp(a) in increasing ASCVD risk can be factored into physician and nurse prescribing practitioners decision making to achieve target lipid levels.

We have previously observed (unpublished observations) that full lipid profiles are frequently measured in primary care about four times more often than in the hospital setting, which contrasts with the low numbers of Lp(a) requests from primary care (5%) in our study. This obviously reflects less awareness about Lp(a) screening in the community setting which attests for the need for broader risk factor education in primary care. This is important given that even in this small sample of the community (i.e. primary care requests), there was indeed a large proportion of individuals who had high-risk Lp(a) levels (26%).

There were a number of limitations in our study. Notably, the assignment to being native Irish was based on surnames only, sourcing online data or authors’ knowledge. Moreover, we had no information regarding the use or not of lipid lowering therapy which could influence the relationship with the lipid subgroups and an 8.5 to 24% increase in average Lp(a) levels in those receiving statin treatment [24].

Knowledge of the true Lp(a) distributions in the Irish population would require a general population study. Moreover, further prospective analyses regarding the influence of cholesterol lowering therapies on Lp(a) serum levels and the rates of major adverse cardiovascular events could be a potential research interest.

## Conclusions

In summary, our study highlights the largest ever cohort study of Lp(a) distribution in an Irish population attending a specialist cardiology service. Lp(a) serum levels in our population were positively skewed which is not dissimilar to other caucasian European cohorts. While these results are not entirely reflective of the general population, they do highlight the very high levels of Lp(a) in our patients attending hospital cardiovascular disease services. Given our repeated poor performance in achieving guideline directed lipid targets [22, 23], this study brings to the fore the additional hidden risks in our population not achieving lipid targets. More education is needed to increase the use of Lp(a) measurements to guide better lipid target achievement particularly in the primary care settings [22].
